# Thermoplastic polyurethane flexible capacitive proximity sensor reinforced by CNTs for applications in the creative industries

**DOI:** 10.1038/s41598-020-80071-0

**Published:** 2021-01-13

**Authors:** Reza Moheimani, Nojan Aliahmad, Nahal Aliheidari, Mangilal Agarwal, Hamid Dalir

**Affiliations:** 1 Integrated Nanosystems Development Institute (INDI), Indiana University-Purdue University Indianapolis, Indianapolis, IN 46202 USA; 2 School of Mechanical Engineering, Purdue University, West Lafayette, IN 47907 USA; 3 Department of Mechanical and Energy Engineering, Indiana University-Purdue University Indianapolis, Indianapolis, IN 46202 USA

**Keywords:** Electrical and electronic engineering, Mechanical engineering, Engineering, Materials science, Nanoscience and technology, Physics

## Abstract

Wearable sensing platforms have been rapidly advanced over recent years, thanks to numerous achievements in a variety of sensor fabrication techniques. However, the development of a flexible proximity sensor that can perform in a large range of object mobility remains a challenge. Here, a polymer-based sensor that utilizes a nanostructure composite as the sensing element has been presented for forthcoming usage in healthcare and automotive applications. Thermoplastic Polyurethane (TPU)/Carbon Nanotubes (CNTs) composites are capable of detecting presence of an external object in a wide range of distance. The proximity sensor exhibits an unprecedented detection distance of 120 mm with a resolution of 0.3%/mm. The architecture and manufacturing procedures of TPU/CNTs sensor are straightforward and performance of the proximity sensor shows robustness to reproducibility as well as excellent electrical and mechanical flexibility under different bending radii and over hundreds of bending cycles with variation of 4.7% and 4.2%, respectively. Tunneling and fringing effects are addressed as the sensing mechanism to explain significant capacitance changes. Percolation threshold analysis of different TPU/CNT contents indicated that nanocomposites having 2 wt% carbon nanotubes are exhibiting excellent sensing capabilities to achieve maximum detection accuracy and least noise among others. Fringing capacitance effect of the structure has been systematically analyzed by ANSYS Maxwell (Ansoft) simulation, as the experiments precisely supports the sensitivity trend in simulation. Our results introduce a new mainstream platform to realize an ultrasensitive perception of objects, presenting a promising prototype for application in wearable proximity sensors for motion analysis and artificial electronic skin.

## Introduction

Electronic technologies including sensors are one of the key components in smart devices. Flexible sensors have been highly explored recently for incorporation into textiles or for direct connection to the body of a human/robot for wearable smart devices^1–6^ and human robotic systems^7–9^. Furthermore, flexible sensors can be implemented as artificial skin and medical prosthetic, chiefly delivering a sensing interface while transmitting information to prevent from damage between human beings and robots as well as the nature^10–12^. Multiple flexible or stretchable sensors have been developed by Micro-electro-mechanical system (MEMS) micromachining techniques for different purposes. For instance, strain sensors^13–15^ can detect body motion, tactile sensors^16–22^ enable to monitor three-axis handling/manipulation of objects, while proximity sensors^20,21,23,24^ avoid any possible accident of humans and robots to unknown obstacles. Among them, proximity sensors are extremely appealing candidates for nondestructive realizing of collision prevention in industry. Thereafter, it is necessary to be able to detect the presence of the object without making contact. To benefit the contactless measurement, studies have been extended on integration and incorporation of proximity sensing function in many electronic platforms. A proximity sensor often looks for the change in the field of either electromagnetic or electrostatic, whose sensing techniques are such as ultrasonic, optical, magnetic induction, and capacitive measurement^25–29^. Capacitive proximity sensors (CPSs) have been widely deployed for their advantages over other sensors thanks to their lightweight, relatively economical, fast detection of a wide range of materials, and readily embedded on both flat and curved working substrates (flexible and variable structure design). To date, the reported target objects for capacitive proximity sensors have been restricted to human finger and conductors, with limited distance resolution^30,31^. These sensors are typically based on metal and silicon substrates in a simple printed circuit board (PCB), constituting a number of circuits and complex layered matrix arrays. Other limitations are addressed such as being too brittle to endure large deformation and not flexible enough to cover curved surfaces. Therefore, flexible and conductive materials are required in CPSs. Accordingly, sensor dielectric layer is required to be fabricated using various electronically conducting polymers, elastomers with low modulus, such as poly ethylene terephthalate (PET), Polyimide (PI), or Polydimethlysiloxane (PDMS)^32–34^. These flexible polymers are frequently proposed as the composites substrates for preparation of resistive and capacitive sensors/arrays^35–39^. Furthermore, the development of flexible conductive electrodes is reported to be essential for increasing the sensitivity of flexible capacitive sensors. Accordingly, metal nanowires/nanoparticles^23,38,40,41^, carbon nanotubes CNTs^42–47^, and graphene^30,48^ have been widely explored as the conductive layers/electrodes. Although metal interdigitated electrodes have been designed and engineered on polymer substrates to employ flexible capacitive micro-sensors, interfacial adhesion between the metal electrodes, polymer and electrode on the film are reported to be very challenging^32,40^.

Among the three dominant emerging nanoscale materials addressed in the literature, CNTs, as active sensing elements, have been the center of attention as an alternative to conventional materials because they have remarkable interfacial, mechanical and electrical properties^52–55^. For instance, elastomer composites with incorporation of CNTs show great potential with promising features for electronic device platforms^42,56^. Recently, vertically aligned CNTs (VACNTs) have been modeled as interdigitated electrodes on a silicon substrate for capacitive sensing^57^. CNTs have also been utilized as the sensing nanofillers for providing conductive polymer composites, which can eliminate the possible interfacial adhesion issues and crack propagation problems of metal film layers patterned on polymer substrates^33^. Different processes have been typically employed to fabricate CNT–polymer nanocomposites sensors, such as mechanical stirring, vacuum filtration, nanoimprint lithography and inkjet printing^55^; however, shaping CNTs in an uniform line pattern as sensing elements are reported to be very complex by these methods^58,59^. Although CNT can provide unique properties to a polymeric structure, it is still a challenge to integrate CNTs within the structure for further applications^60–62^. Furthermore, numerous processes of polymer micromachining have been newly developed to employ in polymer-based flexible sensors^63,64^.

On the other side, most measurements in CNT-based capacitive sensor industry are being focused on a deformation-based nature (pressure/strain sensors); however, this paper intends to investigate more on proximity distance measurement. To benefit the contactless measurement, a couple of studies have been carried out on incorporation of nanofiller based proximity sensing function to the applications for other types of sensors like, tactile, pressure and strain sensors^23,28,49–51,65,66^, becoming an increasing prevalence in wearable electronics. The details of main features of recent capacitive proximity sensors (CPSs) deposited on different flexible polymeric substrates with nanostructured particles/fillers are discussed in the Table 1. As indicated, there are only very few numbers of flexible nanocomposite polymeric CPSs with a large range of detection.

**Table 1:** Review of main characteristics of flexible capacitance-type proximity sensors reinforced by nanomaterials.

Active materials and substrate	Sensitivity $\left[{\%}\frac{\Delta \text{C}}{{\text{C}}_{0}}{\text{m}\text{m}}^{-1}\right]$	Response time (1 pF)	Resolution	Size/shape (area)	Operational range	Other industrial features
Graphene/PET/acrylic^30^ PET (mesh-structured) Graphene (electrodes) Acrylic polymer (dielectric layer)	$0.67$ (iron) 0.11 (finger)	< 60 ms	5 mm	4 × 6 cm^2^ 8 × 8 array (64 channels) Thickness 0.03 mm	10 mm (iron) 70 mm (finger)	Touch sensing Searchability ~ 8–15% (${r}_{b}$ =1.5 mm)
PDMS/AgNWs/PET^23,38,40^ PDMS (dielectric layer) AgNWS (electrodes)	0.06–0.12 (finger)	< 40 ms	5 mm	2.5 × 7.5 cm^2^ Thickness 1 mm	90–140 mm	All pressure sensing Reversibility^38,40^ (up to 100 kPa) and (50% strain)^38^ Durability (200 cycles for 100 kPa)^40^ Stability (2 h)^23^ Bending stability (300 cycles and ${r}_{b}$ =30 mm)^23^
CMC/MWCNT/silicone^49,50^ CMC (elastomer composite sheet)	0.10 (copper)	–	2 mm	3.2 × 3.2 cm^2^ FPCB electrode layer Thickness 0.6 mm	60 mm	Inductive and capacitive sensing modes Repeatable and reversibility (5cycles)^50^ Durability (3000 cycles for 150 kPa)^50^ CMC (0%–1.5%–3%–5%–8%) Maximum detection 1.5%^49^ and 8%^50^
CNC/m-rGO/epoxy^51^ GO (conducting particles)	7.8 (finger) zero (copper and plastic rod)	–	0.5 mm	2 × 1 cm^2^ Thickness 0.16 mm	6 mm	Touch sensor Durability (100 cycles at the distance of 0.2 mm) Good stability, high reproducibility Suitable recovery time (3 s)

PET: Ultrathin Polyethylene Terephthalate, FPCB: flexible printed circuit board, CNC: cellulose nanocrystal, GO: Graphene Oxide, PDMS: polydimethylsiloxane, AgNWs: silver nanowires, CMC: carbon microcoils, MWCNT: multiwall carbon nanotube, ms: millisecond.

This study offers an innovative capacitive proximity sensor enabling sensing objects within a wide range. The nanocomposite ultrasensitive proximity sensor has a simple nanostructure compared with previous studies, but with complex microstructure in which CNTs are melt-mixed in the mold substrate thermoplastic polyurethane (TPU). For the first time, the effect of CNT content is studied on the proximity sensitivity of the flexible capacitive sensor. The sensitivity distribution of the sensor is simulated with Finite Element Modeling (FEM) electrostatic simulation software, and thus, voltage distribution and capacitance reduction behavior are also being examined in the following experiments. The current proximity sensor leaps ahead of the currently used CPSs with their limited sensing distance and provides users with a direct, quick, and easy manufacturing way to interact based on electromechanical principles.

## Materials and method

### Materials preparation

**Figure 1:**
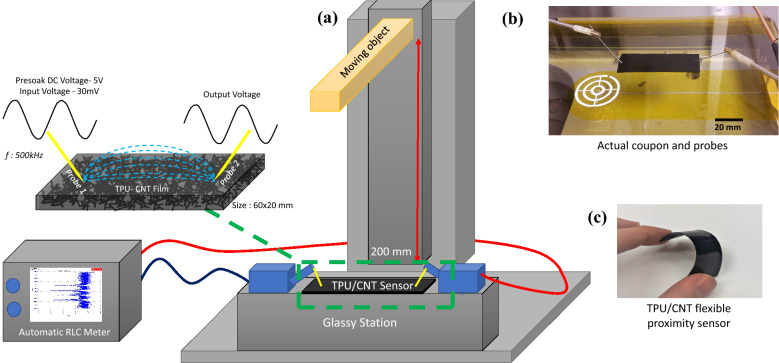
Schematic illustration of a TPU/CNT proximity sensor setup. (**a**) Distances ranging from 220 to 20 mm were applied by using a probing station (Keithley 4200-SCS, Tektronix, USA). The film was fixed over a glassy substrate to eliminate the noise and the sensing object (brass bar-10 × 20 × 200 mm) approached the sample with a speed of 6.6 mm/s (Video S1). To detect the maximum change in the capacitance, the samples were pre-soaked with 5 V direct current (DC) to saturate and reduce the tunneling effect. Furthermore, a 30 mv alternate current (AC) swiping signal was applied to measure the capacitance of the film with varying frequencies. Negative change plot in capacitance showing sensitivity on RLC meter screen. (**b**) The sensor probes were mechanically semi-planner with an angle of 45° to reduce noise and the penetration depth inside the film. (**c**) Sensor being bent to show the thickness and the flexibility.

TPU (Elastollan 1185A polyurethane, with a density of 1.12 g/cm^3^) and TPU/MWCNT masterbatch (A 5 wt% of TPU/MWCNT containing NC7000 MWCNT with 90% purity with an average diameter and length of 9.5 nm and 1.5 μm, respectively) were purchased and diluted. TPU filaments containing 1, 2, 3, 4 and 5 wt% MWCNT were produced by diluting a 5 wt% masterbatch of TPU/MWCNT with pure TPU through a 16 mm twin-screw extruder with L/D ratio of 40 (LabTech Engineering Company LTD., Thailand). To make TPU-CNT films, pellets of TPU-CNT were then cut from filaments and later compressed in a hot press machine (Carver Inc., Wabash, Indiana) at 2.25 metric tons at 185 °C for 60 s. The films were cooled down to the room temperature and utilized to fabricate the sensors and for characterizations. Samples were cut from the films in 60 × 20 mm squares with 0.5 mm thickness. Figure 1 showed the final product and the flexibility of the film sensor along with the experiment setup.

### Proximity measurements and sensor readout

Distances ranging from 20 to 220 mm were applied by using a probing station (Keithley 4200-SCS, Tektronix, USA). The TPU-CNT film was fixed over a glass substrate to eliminate noise and the sensing object (brass bar—10 mm (height) × 20 mm (width) × 200 mm (length)) started approaching the sample with a speed of 6.6 mm/s after 60 s (Video S1). The impedance analysis was performed by the modular instrumentation using a probing station. To detect the maximum change in the capacitance, the samples were pre-soaked with 5 V direct current (DC) to saturate and reduce the tunneling effect and also to polarize the polymer and form the surface charge. Furthermore, a 30 mv alternate current (AC) swiping signal was applied to measure the capacitance of the film with varying frequencies to achieve maximum stability of the working window for the fabricated sensors. As demonstrated in Fig. 1a,b, the experimental setup and how change of capacitance to the initial capacitance ($\Delta C/{C}_{0})$ were analyzed. The sensor probes were mechanically co-planner with an angle of 45° to eliminate the noise and reduce the penetration depth inside the film. The entire three set of tests were done for each CNT content. Other conditions, including temperature and humidity, were strictly controlled to obtain a precise measurement.

### Electrical testing

To investigate the percolation behavior of the bulk TPU/CNTs nanocomposites, the in-plane conductivity of hot-pressed nanocomposites was measured on 60 mm by 20 mm square samples with 0.5 mm thickness. The narrow strip of two ends of samples were then coated with CircuitWorks CW2400 conductive silver epoxy and the bulk electrical conductivity was calculated by measuring the resistance using Keithley 2400 4-probes. Electrical conductivity of each sample is being calculated by using: *σ* = *L*/*RS*, where *σ* is the volume conductivity, R is the volume resistance, S is the cross-section area of the strip, and L is the length between the electrodes.

### Finite element analysis

3D and Finite Element Modeling were performed using electromagnetic field simulation software (Ansys Maxwell 2018). Material properties with affiliated modules including relative permittivity and bulk conductivity of 2 wt%. TPU/CNT sample were experimentally measured and assigned to the software. Furthermore, boundary conditions, vacuum domain (15 cm × 10 cm × 10 cm) and excitation (i.e. applied voltages) are implemented, accordingly. Electric field streamlines in the vacuum domain on the symmetric plane passed through, where a voltage difference of 5 V is applied between the two probes of tungsten (the driving and the sensing electrodes). Maximum length of the element with an efficient computational time was optimized about 1 mm. Regarding mesh objectivity, the mesh was refined about 30% in every run for ten iteration. The mesh density is set to be “extremely fine”; and the mesh number ends up in 302,982.

## Results and discussion

**Figure 2:**
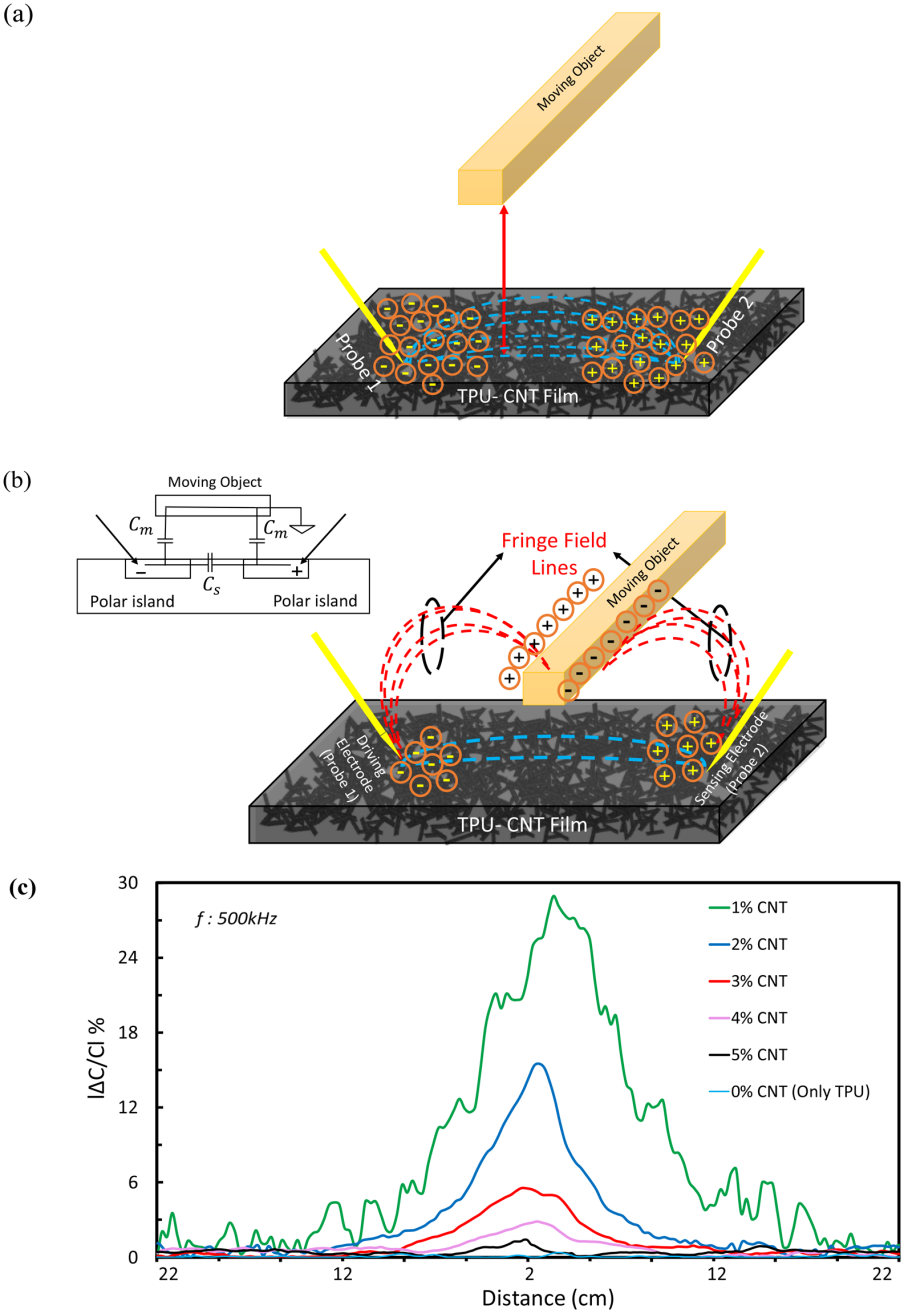
Electrical field lines of proximity sensor and maximum sensitivity. (**a**) Moving object at the farthest distance from sensor (i.e., 220 mm), no electrical field lines exist between probs and object. Bent electric filed lines (self-capacitance) are shown. (**b**) Moving object at the closet distance to sensor (i.e., 20 mm), mutual capacitance becomes clear. Shunting of the initial electeric lines causes very strong and scattered fring field between object, film and probes, and capacitance drops radically. (**c**) Maximum sensitivity comparison of different wt%. CNTs. Absolute percentage change of capacitance to the initial capacitance is plotted against distance.

TPU-CNT films can be a replacement for conventional proximity sensors, due to its simpler fabrication process, flexibility, and durability. Proximity detection is defined by the change in the amount of measured capacitance of the TPU-CNT film due to the presence of an object in the electric field around it. Figure 1a–c illustrates the measurement setup and how data was analyzed. The sensor probes are mechanically semi-planar with an angle of 45° to eliminate the noise and reduce the penetration depth inside the film. To identify physics of the sensation, this sensing film constitutes two types of capacitors including a capacitor between nearby probes (self-capacitance—${C}_{s}$) and fringe capacitance) mutual capacitance—${C}_{m}$), caused by the overlapping fringing field between the surface of the object, film and probes. As shown in Fig. 2a, the electric field line does not fully horizontally match with the linear distance between the probes. When an object is not close to the film, the fringe capacitance between each probe with object is negligible. At closer distances to the probes, the fringe field between film and the sensing object becomes significant, as seen in Fig. 2b. Theoretically, shunting of the electric field changes the overall capacitance of the film. Gauss' law for electricity states that the electric flux through any closed surface is proportional to the total charge enclosed by the surface, $\int \overrightarrow{E} \cdot d\overrightarrow{A}=\frac{\text{Q}}{{\varepsilon }_{0}}$.

Hence, electric field strength emanating from film capacitor is shunted and weakened by the object, thereby lowering the charges stored in the film capacitor. Furthermore, the mutual capacitance of sensor increases due to the reduced distance between probes and object while the capacitance of the actual film (self-capacitance) drops due to the change in the charge balance (C = Q/V). Thus, while right probe (sensing electrode) and the moving object are DC grounded, certain amount of charges migrates from the grounded probe to the moving object, resulting in the reduction of stored surface charges on the film and subsequently overall capacitance reduction (Fig. 2a,b).

Having an optimal nanocomposite sensor, five different CNT contents are studied. Figure 2c shows sensitivity (ΔC/C%) of proximity sensor with respect to the weight percent of CNT. However, Pure TPU does not show any noticeable capacitance change under the test. Absolute percentage change of capacitance to the initial capacitance was plotted against distance. Measurement is aimed to be performed within a range of 20 cm. The object starts moving closer to the sensor from a distance of 22 cm and eventually stops off at 2 cm. Due to the surface charge migration (fringe effect), the capacitance radically drops for the distances lower than 2 cm. Below 2 cm, sensor acts more like a tactile capacitive sensor which is not the purpose of this study. Although changes in the capacitance occur relatively from 22 cm for CNTs 1 wt%, the limit for visible proximity sensing is about 12 cm, which becomes prominent at 2 cm. We also directly calculated the sensor’s sensitivity to the detection distance. Our calculations suggest that the sensor’s capacitance output will vary on the order of 10 fF per centimeter. Given that the sensor operates on the sensitivity order of 100 fF, this represents a sensitivity of 0.3%/mm, meaning that it should be fairly sensitive to distance variation in a long detection range. The response time of this sensor is about 30 ms, which is comparable with reported values in Table 1.

**Figure 3:**
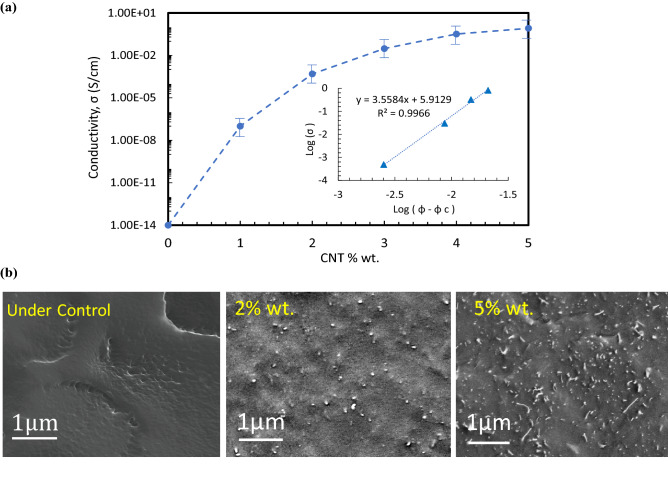
Electrical conductivity and CNTs distribution. (**a**) Electrical conductivity of TPU/CNT samples vs. CNT content wt% are employed to do calculation of percolation threshold. (**b**) SEM distribution images of cross-section fracture surface of the neat, 2 wt% and 5 wt% MWCNTs in TPU nanocomposite. Bright dots and lines are the carbon nanotubes within the polymer matrix.

The improved sensitivity of the very sensor possibly arises from the nanostructured architecture, which have a higher density of the electric field originating from larger polarization of the polymer by the presence of highly conductive nano tubes. Thus, the field can be enhanced while the nano particles are acting as an embedded conducting network within the polymer structure and it leads to improvement of polarization inside the nonconductive polymer. As seen from Fig. 2c, the sensitivity graph shows 2 wt% with quite high sensitivity but less noticeable noise among other CNT contents. The physics behind the observed noise data (Fig. 2c) is that this number of nanoparticles provides a certain number of conductive pathways due to the percolation of CNTs inside the TPU structure. In percolation threshold, not only the electrical conductivity of the composite enhances remarkably but it also rises the chance of trapping charges by applying a presoak voltage. Hence, the conductivity of the film can be generally described in terms of modified classical percolation behavior using eq (1):

1 $$\sigma ={\sigma }_{0}{\left(\varnothing -{\varnothing }_{c}\right)}^{t}$$

where ${{\varnothing }}_{c}$ is the percolation threshold and *t* is the exponent values, which depends on the dimensionality of the composite. Fitting the film conductivity data with Eq. (1). yields a percolation threshold. As shown in Fig. 3a, the exponent t is 3.55 and ${\sigma }_{0}$ is 5.9 from the fitting. Calculation of percolation threshold resulted in 0.96% volume which is about 1.3 wt%. The fitted value for the critical exponent goes away from the theoretical universal scaling value t = 2. The high exponent value can be attributed to a tunneling percolation network. This can be explained by multiple percolations in conducting polymer systems, as proposed by researchers^67,68^ such that the electrical conductivity of a polymeric system is determined by the morphology of filler particles in addition to filler loading. In the case of tunneling percolation network, such a large exponent indicates a broad distribution of the tunneling resistance and hence a large distribution of the inter particle distance. In addition, the films containing CNT alone are observed to show a rapid increase in electrical conductivity by about 7 orders of magnitude (from 5.06 × 10^-14^ to 1.2 × 10^-7^Scm−1) when the CNT content was increased from neat to 1 wt%. This sharp change of conductivity indicates the formation of percolating network in the polymer matrix.

Figure 3b shows cross-section microstructure of TPU-CNTs by Scanning Electron Microscope (SEM) for 0 wt%, 2 wt% and 5 wt%. CNT contents. Bright dots and lines are the carbon nanotubes within the polymer matrix. It has been observed that by the melt-mixing method, CNTs are homogeneously dispersed in the polymer matrix. Furthermore, percolation process shows that conductive pathways are fully constructed at the value of 1.3 wt%. It can be concluded that since the conductive pathways become solid and significant after the percolation threshold, not only lower standard deviations (i.e., noise) but a good sensitivity also are expected.

Additionally, another remarkable trend is that proximity sensitivity of the TPU-CNT sensors lowers by increasing the CNT content (from 1 wt% toward 5 wt% CNTs). TPU has been known as a high polar polymer which can be used as a perfect dielectric. TPU in the form of multi-block copolymers also contains of more high polarity segments (called hard-short segments), which leads to formation of surface charge and consequently higher polarization in the presence of an electric field. Addition of CNTs inside the TPU structure, makes the film semi-conductive and increases the polarity. As consequently observed, the initial capacitance of the TPU/CNT film has been increased by adding CNTs content. However, the maximum sensitivity (ΔC/C%) has been observed at the lower content of CNTs (1 wt%). This phenomenon allows us to conclude that the maximum sensitivity would be possibly achieved around the percolation threshold (1.3%wt CNT) and afterward, the sensitivity drastically drops. This decreasing trend can be interpreted by the performance of both matrix and nanoparticles. By addition of CNT, clusters of non-conductive TPU and highly conductive CNT form higher capability of polarization. Consequently, adding higher percentage of CNT^69–72^ increases the conductivity and initial capacitance, but lowers slope of polarity growth. Within higher CNT concentrations, larger polar islands are being formed on the TPU film in presence of an electric field (Fig. 2a). Thus, by approaching the object with limited charges (fringe field-Fig. 2b) to the TPU-CNT samples with higher polarity, less percentage change of capacitance has been measured due to less difference in the transferred charges. This supports the observation of less sensitivity in samples with higher CNT concentration.

**Figure 4:**
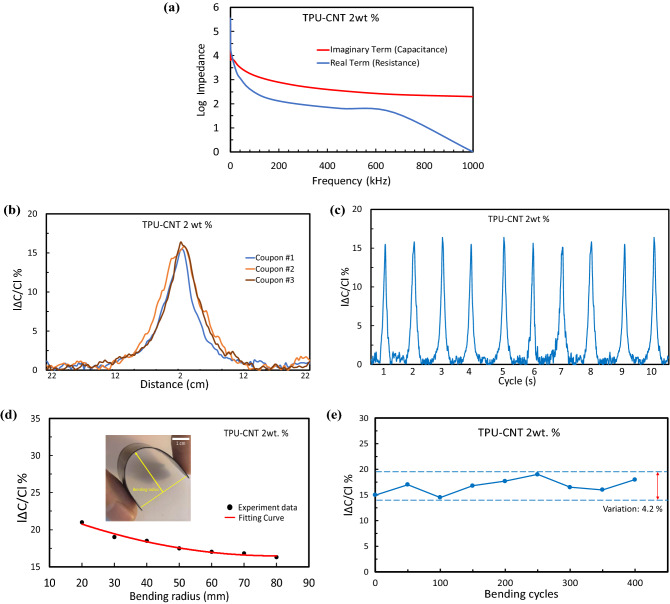
Sensor characterization with 2 wt%. CNT (**a**) Nyquist stability plot of the impedance sensing to the magnetic field at different exciting frequencies. From EIS, the stable region of frequency response for imaginary and real are initiated around 500 kHz. (**b**) Reproducibility response of sensors for three different coupons of similar manufacturing process. (**c**) Repeatability response with several identical approaching cycles along with repeatable peaks. (**d**) Relative change in capacitance of the sensor film under various bending radii of 20–80 mm. (**e**) Mechanical flexibility of the tactile sensor—relative change in capacitance measured for 400 bending/relaxing cycles at a bending radius of 20 mm.

To achieve the maximum stability for the capacitance measurements, in Fig. 4a, Nyquist plots were utilized to demonstrate the impedance sensitivity with respect to the frequency for TPU-CNT 2 wt% sample as to determine the stable region of frequency. As it can be observed from Electrochemical Impedance Spectroscopy (EIS), the stable region of frequency response for imaginary impedance (capacitance) is initiated at around 500 kHz. On the other hand, that of the real impedance (resistance) is detected between 400 and 600 kHz. Therefore, to achieve the maximum stability and efficiency, 500 kHz was chosen the sensor operating frequency. This frequency provides maximum stability for the impedance while the frequency response does not alter the resistance of the film or change the capacitance of the sensors. This would improve in minimization of measurement errors (i.e., noise) and provide maximum sensitivity for the film sensor. Despite higher frequencies may render more sensitivity and relatively lower noise, energy consumption drastically increases. Reproducibility and repeatability sensing responses of 2 wt%. CNTs are shown in Fig. 4b,c. Reproducibility response of the sensor is assessed for three different coupons of similar manufacturing process. Subsequently, cycling response has been analyzed and its peaks are apparently very self-repeating (Fig. 4c).

An important improvement of polymeric proximity sensors, in order to widen their applicability, is sufficient mechanical flexibility to allow for stable operation under bending conditions. Figure 4d shows a photograph of the flexible proximity sensor subjected to bending. The flexibility of the sensor was examined by measuring capacitance changes with respect to its before-bent sample under applying various bending radii. The capacitance increases as the bending radius decreases and reaches ≈ 21% at a maximum bending (radius of 20 mm). At the lower bending curvature (higher radii) of 2 wt% TPU-CNT sensor, the percentage magnitude of capacitance change becomes closer to the before-bent one (≈ 15%)—shown earlier in Fig. 2c. So, the capacitive response of highest radius (lowest external bending) is insignificant (4.7%) respect to that of lowest radius (highest bending). In another word, the capacitance recovered to its initial value with no permanent changes, demonstrating robust mechanical flexibility of the TPU-CNT sensor. To evaluate the durability of the sensor against bending, we conducted cyclic tests with repeated bending and relaxing (see Fig. 4e). The variation of the capacitance change is lower than 4.2% over 400 cycles of bending at a radius of 20 mm, and thus the capacitive output of the sensor remained stable to the repeated bending.

**Figure 5:**
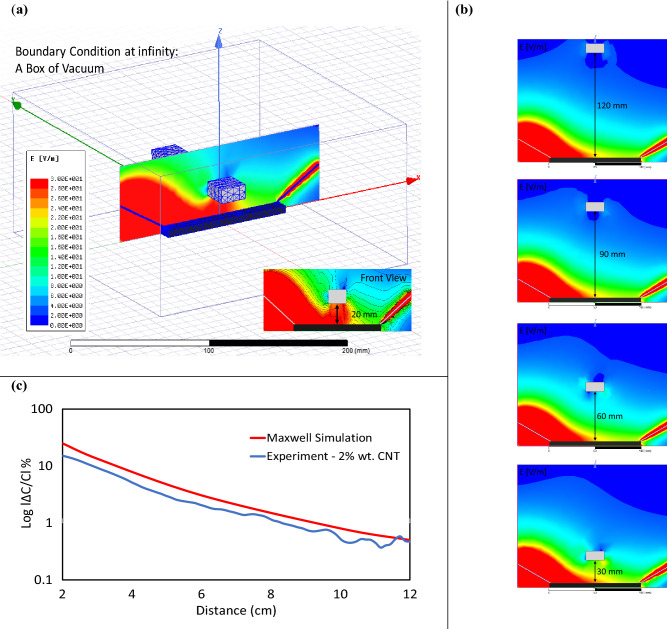
Schematic representation of simulation results of the basic fringe field cell. (**a**) A simple 3D model in Ansoft Maxwell along with implemented material properties, boundary conditions, vacuum domain, mesh and voltage distribution of sensor at closest proximity to the sensor (20 mm) inside a vacuum box. (**b**) Voltage distribution between two probes in range of 120 mm to 30 mm. Capacitance between two probes is mainly determined by fringe effects. (**c**) Experimental and Maxwell simulation comparison of capacitance change as a function of distance.

As earlier mentioned, the sensing mechanism is based on the object interference with the electrical field between two probes. The interference disturbed and reduced the number of electric field lines (i.e., self-capacitance) and consequently, stored charged decreased radically on the polymer. To assure that the capacitance change comes from the fringe phenomenon, a simple 3D model is simulated in Ansoft Maxwell. Figure 5a demonstrates the measurement setup in which object, material properties, boundary conditions, vacuum domain and excitation (i.e. applied voltages) are implemented (further details are available in FEM “Materials and method” section). This figure shows the electric field streamlines in the vacuum domain on the symmetric plane passed through, where a voltage difference of 5 V is applied between the two probes of tungsten. Primitivity and bulk conductivity of 2 wt%. CNT sample are approximately employed as the material input property of TPU-CNT. Capacitance measurements have been illustrated in Fig. 5b, for different distances between the grounded object and the substrate (i.e. electrostatic simulation). Applying the voltage to the TPU-CNT film sensor, the formation of potential gradients around the substrates is observed. By introducing the object inside the formed electric field, the disturbance of the field has been detected and used as the sensing mechanism. As observed in Fig. 5b, while the object is at very far distance (> 80 mm), there is minimal change in the potential surfaces (stored charges) and the field is uniform. By lowering the distance, the field shape changes and high potential surface forms between the object and the surface of the film in z direction and thus leads to the migration of surface charges and formation of fringe fields around the object. Although the object is smaller than the TPU-CNT sensing film, the fringe fields have been created on both extremities of the object. The fields change the polarization (the charge balance) of the TPU-CNT film and outcome in formation of unexpected store charged between the object and the film. By reduction of the distance (< 30 mm), the field creates a new capacitor between the film and the object and the stored charge on the film are being interrupted, and this eventually leads to more reduction in the capacitance of the film sensor. While the electric field between the probes and the object (mutual capacitance-Cm) are responsible for the reduction of electrodes capacitance (self-capacitance), their change is not being measured neither in the simulation model nor in the experiments. In a short, the overall capacitance changes of the sensor (self-capacitance) is majorly under the influence of object movement and the resistivity of film sensor. As displayed in Fig. 5c, capacitance decreases when the object-sensor distance goes from 120 to 20 mm. Furthermore, the experimental result closely follows the Maxwell simulation trend in logarithmic scale and simulation is capable of identifying the fringing effect.

## Conclusion

A flexible ultrasensitive polymer-based proximity sensor was presented. The TPU was reinforced by adding CNTs and delivered the potential to adjust the sensitivity based on electrical behavior of CNTs. The developed sensor was sensing based on the change of the capacitance that was mainly quantified by fringe effects. Although 1 wt%. CNTs showed highest sensitivity, 2 wt% CNTs earlier indicated as percolation threshold exhibited lowest noise along with significant sensitivity. Fringing field and tunneling effects (polarity) were addressed as two main explanations of observing the changes trend of the capacitance(sensitivity). As the object approached the sensor, electric field partially started to be distracted and accumulated over the object. Local surface charges migrated from the film sensor and consequently the self-capacitance declined. Additionally, tunneling network pathways resulted in higher amount of polarization (less transferred charge) justified higher sensitivity in the samples with lower wt% of CNTs. Frequency of 500 kHz was selected as the optimum to minimize noise as well as power consumption. We further discussed extraordinary flexibility under the bending loading and durability during loading/unloading process with the variation of below 5%. The sensor can sense the object movement with a fast response time/range, high repeatability and cycling feasibility.

Lastly, the numerical simulation results were capable of accurately following the fringe effect and showed decreasing trend of capacitance as previously shown in the experiments. Overall, the detection performance of our described proximity sensor implies potential applications in the field of smart flexible devices which is the best platform to realize and monitor the movement of objects. The flexibility, high sensitivity, and notable stability of the proposed sensor, along with extremely simple fabrication process, offer an alternative to the current controlled electrical proximity sensors for incorporation into wearable gadgets and future smart electronic devices.

## Supplementary Information

Supplementary Video Legend.

Supplementary Video S1.
