# Supplementary material for: Thermoplastic polyurethane flexible capacitive proximity sensor reinforced by CNTs for applications in the creative industries
Source: Sci Rep. 2021 Jan 13;11:1104. doi: 10.1038/s41598-020-80071-0 (PMC7806639; doi:10.1038/s41598-020-80071-0)
Supplement: Supplementary file 1 — Supplementary Video Legend. [file 41598_2020_80071_MOESM1_ESM.docx]

Thermoplastic Polyurethane Flexible Capacitive Proximity Sensor Reinforced by CNTs for Applications in the Creative Industries

**Reza Moheimani, Nojan Aliahmad, Nahal Aliheidari, Mangilal Agarwal and Hamid Dalir**

Video shows the proximity sensing. As requested, in this video we tried to just exhibit how the measurement has been done. In the original test, we carried out the tests with a lifter, scale, and station.
